# Curcumin-Arteether Combination Therapy of *Plasmodium berghei*-Infected Mice Prevents Recrudescence Through Immunomodulation

**DOI:** 10.1371/journal.pone.0029442

**Published:** 2012-01-20

**Authors:** Palakkod G. Vathsala, Chaitanya Dende, Viswanathan Arun Nagaraj, Debapriya Bhattacharya, Gobardhan Das, Pundi N. Rangarajan, Govindarajan Padmanaban

**Affiliations:** 1 Department of Biochemistry, Indian Institute of Science, Bangalore, India; 2 International Centre for Genetic Engineering and Biotechnology, New Delhi, India; Weill Cornell Medical College, United States of America

## Abstract

Earlier studies in this laboratory have shown the potential of artemisinin-curcumin combination therapy in experimental malaria. In a parasite recrudescence model in mice infected with *Plasmodium berghei* (ANKA), a single dose of alpha,beta-arteether (ART) with three oral doses of curcumin prevented recrudescence, providing almost 95% protection. The parasites were completely cleared in blood with ART-alone (AE) or ART+curcumin (AC) treatments in the short-term, although the clearance was faster in the latter case involving increased ROS generation. But, parasites in liver and spleen were not cleared in AE or AC treatments, perhaps, serving as a reservoir for recrudescence. Parasitemia in blood reached up to 60% in AE-treated mice during the recrudescence phase, leading to death of animals. A transient increase of up to 2–3% parasitemia was observed in AC-treatment, leading to protection and reversal of splenomegaly. A striking increase in spleen mRNA levels for TLR2, IL-10 and IgG-subclass antibodies but a decrease in those for INFγ and IL-12 was observed in AC-treatment. There was a striking increase in IL-10 and IgG subclass antibody levels but a decrease in INFγ levels in sera leading to protection against recrudescence. AC-treatment failed to protect against recrudescence in TLR2^−/−^ and IL-10^−/−^ animals. IL-10 injection to AE-treated wild type mice and AC-treated TLR2^−/−^ mice was able to prolong survival. Blood from the recrudescence phase in AE-treatment, but not from AC-treatment, was able to reinfect and kill naïve animals. Sera from the recrudescence phase of AC-treated animals reacted with several parasite proteins compared to that from AE-treated animals. It is proposed that activation of TLR2-mediated innate immune response leading to enhanced IL-10 production and generation of anti-parasite antibodies contribute to protective immunity in AC-treated mice. These results indicate a potential for curcumin-based combination therapy to be tested for prevention of recrudescence in falciparum and relapse in vivax malaria.

## Introduction

The development of resistance to front-line antimalarial drugs such as chloroquine and antifolates as well as decreased efficacy of mefloquine and even quinine in malaria endemic regions has led to introduction of artemisinin derivatives as the front line drug [1]. Although, artemisinins are particularly more active than any other antimalarial, reducing the number of parasites by approximately 10^4^ per cycle [2], they need to be taken for a seven-day period in monotherapy for complete cure. The difficulty in adherence to this regimen as well as use of suboptimal doses would result in recrudescence and development of resistance and is a major concern [3]. This has led to artemisinin derivatives (ART)-based combination therapies in a three-day course regimen [4]. Among several artemisinin derivatives-based combinations being used or tested, artemether-lumefantrine and dihydroartemisinin-piperaquine combinations are considered as most promising [5], [6]. Our previous studies indicated that curcumin from turmeric has antimalarial activity [7]. Interestingly, curcumin was found to be very effective in combination with ART in preventing parasite recrudescence in mice infected with *P. berghei* (NK 65, non-cerebral strain) for 24 hr [8]. In the mouse model used, arteether treatment alone (AE) was found to result in parasite recrudescence, which was prevented by ART-curcumin (AC) combination treatment. It was of interest to examine the mode of action of AC combination treatment in this regard. The results obtained indicate a unique mechanism of action for this combination, suggesting its potential application to prevent recrudescence/relapse in human falciparum and vivax malaria.

## Results and Discussion

### AC Treatment Cleared the Parasite Faster From Blood but Not from Liver and Spleen

**Figure 1:**
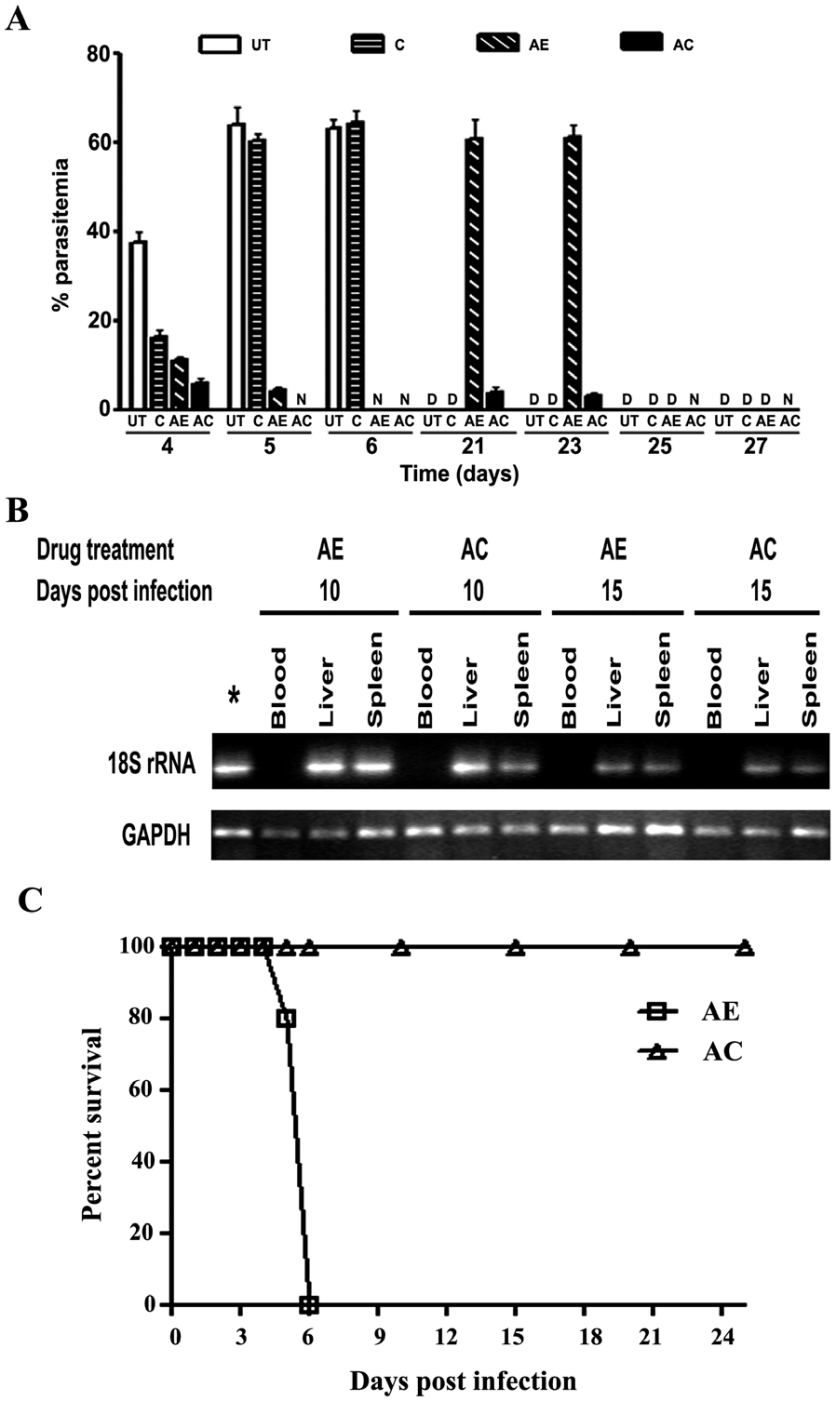
Effect of AE and AC- treatments on parasitemia in mice infected with *P.berghei* for 72 hr. (A) Changes in parasite load in blood at different time periods through real-time PCR analysis of parasite 18S rRNA after drug treatment. UT, untreated; C, curcumin, AE, ART alone; AC, ART+CUR. D, died; N, not detectable. Data provided represent Mean ± S.D. from three animals. The whole series was repeated thrice (total of 9 animals in each group) and number of animals surviving on day 30 in each group were as follows: UT, 0; C, 0; AE, 1; AC, 9. (B) Semiquantitative RT-PCR analysis of parasite 18S rRNA in blood, liver and spleen on day 10 and 15 after infection. GAPDH was used as a control. *, positive control. (C) Effect of injection of blood from recrudescing animals after AE and AC-treatments into naïve animals. Five animals were used in each group.

The mechanism of action of curcumin in the AC combination treatment was investigated in the present study with mice infected with *P. berghei* (ANKA) for 72 hr. Swiss mice were infected with *P. berghei* and after 72 hr given a single injection of ART (750 µg, i.m.) with or without 3 oral doses (5 mg per mouse) of curcumin at 24 hr intervals. To get an accurate estimate of parasitemia, we performed real time RT-PCR with specific primers for parasite 18S ribosomal RNA with RNA from blood [9] and a correlation was established with parasitemia in blood as measured using smear microscopy with Giemsa stain (Figure S1). A semi-quantitative RT-PCR analysis of 18S rRNA with RNA from blood is provided in Figure S2. Results of real time PCR analysis presented in Figure 1A indicate that a parasitemia of around 35% was reached in blood of *P. berghei* infected mice 4 days p.i. These levels increased to around 60% on days 5 and 6 p.i., resulting in mortality by day 5/6 p.i. After administration of one oral dose of curcumin on day 3, the parasitemia transiently decreased to around 15% on day 4 p.i. However, parasitemia increased with the next two doses of curcumin and the animals died between 7 and 9 days p.i. AE and AC treatments resulted in parasitemias of around 10% and 5% respectively on day 4 p.i. and 5% and 0% respectively, on day 5 p.i. On day 6 p.i, no parasite could be detected in blood in either treatment. Thus, parasite clearance in AC treated mice was faster than that observed in mice treated with AE alone. During the period of recrudescence, parasite load shot up to around 57% and 58% on days 21 and 23 p.i. respectively, in mice treated with AE alone and all these animals died during this period. In contrast, a low level of parasitemia of around 3% was seen on days 21 and 23 p.i. in AC-treated animals. However, these parasites were soon cleared and no parasite could be detected in blood on days 25 and 27 p.i. and all these animals survived. A cumulative data of around 400 animals over the last over 4 years indicate that in this recrudescence model, AE-treatment alone was not effective and around 95% of the animals died, conversely AC treatment resulted in close to 95% protection (Table S1). Curcumin was effective only in conjunction with ART, as is also evident from data on only a modest protection obtained with curcumin in ART-resistant, self-clearing *P. chabaudi*-infected mice [10]. However, recent studies have claimed that oral delivery of curcumin bound to chitosan nanoparticles is able to completely cure *P.yeolli*-infected mice [11].

A semi-quantitative RT-PCR analysis for 18 S rRNA indicated that parasite RNA was undetectable in blood, but detectable in liver and spleen on days 10 and 15 p.i. in both AE and AC treated animals (Figure 1B). Thus, AE as well as AC treatments failed to clear parasites in liver and spleen, indicating that these organs could be the reservoir for recrudescence in blood. The survival of rodent malaria merozoites in the lymphatic system was demonstrated by Landau *et al* [12]. Recent studies by Wykes *et al* [13] have shown that *P.berghei* can survive and replicate within CD317^+^ DCs and that a small percentage of these DCs release parasites that can infect erythrocytes in naïve mice. Since, a transient parasitemia of 2 to 3% was seen in AC treatment during the recrudescence phase (Figure 1A), blood from AE and AC- treated animals in the recrudescence phase was injected into fresh animals. The results indicated that while the parasites from the AE treated animals infected and killed the recipients in 5 to 6 days, blood from AC treated animals failed to do so even after several days, indicating that the combination therapy had weakened the parasite, rendering it incompetent to infect fresh cells (Figure 1C).

### Curcumin Levels in Blood and Tissues

Curcumin levels were estimated in plasma, liver and spleen using MALDI TOFF MS at different time intervals [14], [15]. Consistent values were obtained 4 hr after curcumin administration, and representative data are provided after the third oral dose. The results presented in Table S2 indicate that the values ranged between 2 to 4 µg per ml plasma or per g tissue. No curcumin or its metabolites were detectable beyond 8 hr after curcumin administration. Thus, curcumin has a half-life of only a few hours. It is well established that ART derivatives have a half-life ranging from 45 min to a few hours [3], [16].

### AC Treatment Led to Enhanced Reactive Oxygen Species (ROS) Generation in Infected Cells

**Figure 2:**
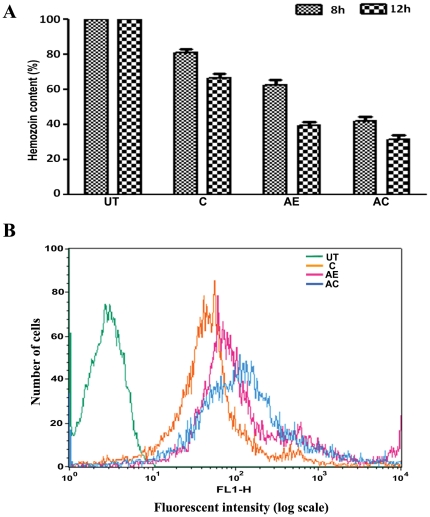
Effect of AE and AC-treatments on hemozoin content and ROS generation in *P.berghei*infected mice. (A). Hemozoin content in parasite 8 hr and 12 hr after a single injection of ART and one oral dose of curcumin. The data represent Mean ± S.D. from three animals. (B) FACS analysis of *P.berghi*–infected red blood cells for ROS measurement 12 hr after drug treatment as per (A).

A general principle in artemisinin-based combinations is that the second drug should have a longer half-life to take care of the residual parasite surviving after the elimination of the primary drug [1], [2]. However, in the case of ART-curcumin combination, both the drugs are of short half life and therefore the mechanisms involved in short-term and long-term effects needed to be evaluated. Curcumin is known to be an anti-oxidant, but can also manifest pro-oxidant activity in presence of metal ions [17]. Cui *et al* [18] have shown that curcumin enhances ROS generation in *P. falciparum* cultures in the concentration range of 20 to 100 µM. Thus, it was of interest to examine the effect of AE and AC treatments on the heme polymerization process giving rise to hemozoin and ROS generation in the parasite. As shown in Figure 2A, curcumin, AE and AC treatments inhibited hemozoin levels, the combination showing maximum effect. While, the inhibition in hemozoin content was more than 60% during the period of experimentation, parasitemia decreased by 10–15%, suggesting that accumulation of heme monomers could be the earlier event. ROS generation was measured in infected blood isolated 12 hr after a single injection of ART and one oral dose of curcumin, by FACS analysis. The formation of intracellular ROS in parasitized red blood cells was measured using 2′,7′-dichlorofluorescin diacetate (DCFH-DA, Sigma- Aldrich) essentially as described by Cui *et al* [18]. DCFH-DA diffuses through the membrane and is hydrolysed by intracellular esterases to DCFH within the cells. DCFH is converted to DCF by the free radicals and its fluorescence is measured by FACS analysis. The results presented in Figure 2B and Table 1 indicate that all the treatments increased mean fluorescent intensity and percent fluorescent cells, with the combination showing maximum effect. These results indicate that the earlier clearance of parasites in blood in AC treatment is, perhaps, mediated by ROS generation in presence of heme-iron.

**Table 1:** Mean fluorescent intensity and percent positive cells in FACS analysis (Figure 2B).

Sample	Mean fluorescent intensity	% positive cells
Control	91.00±11.21	2.05±0.75
Curcumin	230.50±22.56	13.96±1.23
AE	207.82±19.87	18.72±1.98
AE+Curcumin	491.20±31.25	42.49±3.21

Data represent Mean ± SD from three preparations.

### AC treatment Reversed Splenomegaly in *P.berghei*-infected Mice

**Figure 3:**
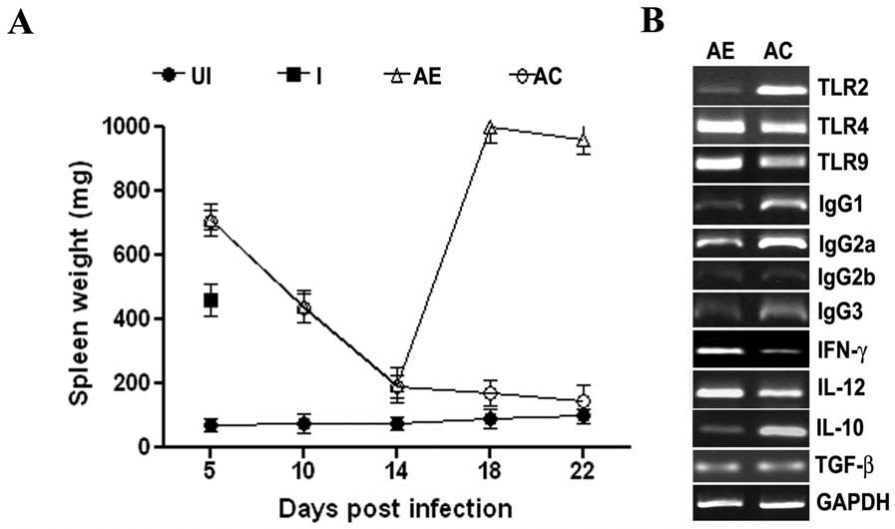
Effect of AE and AC-treatments on changes in spleen mass and mRNA levels of cytokines, TLRs and IgG-sub-class antibodies in *P.berghei*-infected mice. (A) Changes in spleen weight through out the course of infection and drug treatments. Data provided represent Mean ± S.D. from five animals. *, infected animals died 5/6 day (B) Semi-quantitative RT-PCR analysis of spleen RNA on day 14. UI, unifected; I, infected; AE, ART alone; AC, ART+CUR.

RT-PCR data (Figure 1B) clearly indicate that the parasites were not cleared from liver and spleen. Thus, the effect of AC treatment in preventing recrudescence should involve a long-term action of curcumin, mediated through an immune-priming effect. Indication for such action came from the effect of drug treatments on splenomegaly that accompanies malaria. The results presented in Figure 3A indicate that there was a striking increase in spleen weight soon after parasite infection, with a gradual fall with the clearance of parasite load as a result of AE or AC treatment. However, a dramatic increase in spleen weight was evident in AE-treatment after day 14, attesting to parasite recrudescence. This was completely prevented by AC treatment, with the spleen weight tending towards that of uninfected mice.

Thus, it was of interest to look at the arms of innate and adaptive immunity in this experimental set up. TOLL-like receptors (TLRs) of the innate immune system recognize microbial products, including those of protozoan parasites such as *Plasmodium* and activate the development of acquired immune response. The malarial parasite molecules containing pathogen-associated molecular-patterns (PAMPs) include glycophosphosphatidyl (GPI) anchors and hemozoin with associated DNA, which are reported to activate TLR2 and TLR9, respectively. The TLRs are considered to be the central mediators in a pro-inflammatory response in general in malaria, ultimately promoting antibody production, B cell proliferation and T cell differentiation and activation [19]–[21]. However, if the pro-inflammatory response is excessive, over production of IFNγ and TNFα may be detrimental and an association with severe malaria has been shown [22], [23]. Regulatory cytokines such as IL-10 and TGFβ are also produced to suppress excessive proinflammatory responses in human and experimental malaria [24]–[26]. Thus, self-clearing *P. chaubadi AS* [27] and *P. yoelii*-infected [28] mouse models depict the protective role of IFNγ-dependent early innate and cell-mediated mechanisms critical for control of acute stage infection and subsequent antibody production for clearing the chronic phase. On the other hand *P. berghei* ANKA infection in mice, considered as a model for human *P. falciparum* malaria and used in the present study, leads to excessive proinflammatory response and results in early death due to cerebral malaria, requiring the production of regulatory cytokines such as IL-10 to suppress the pro-inflammatory response [24], [26]. Experiments were, therefore, carried out first of all to quantify mRNA levels in spleen for relevant TLRs, pro- and anti-inflammatory cytokines and IgG subclass antibodies. The results presented in Figure 3B indicate that on day 14, AC treatment resulted in a striking increase in TLR2-, but not TLR4- or TLR9- mRNA levels. There was a significant increase in IL-10 mRNA levels, but a decrease in INFγ and IL-12 mRNA levels, compared to AE treatment. TGFβ mRNA levels did not show a change. Interestingly, AC treatment led to an increase in almost all the IgG subclass mRNA levels.

### AC Treatment Supressed INFγ but Enhanced IL-10 Levels in Serum

**Figure 4:**
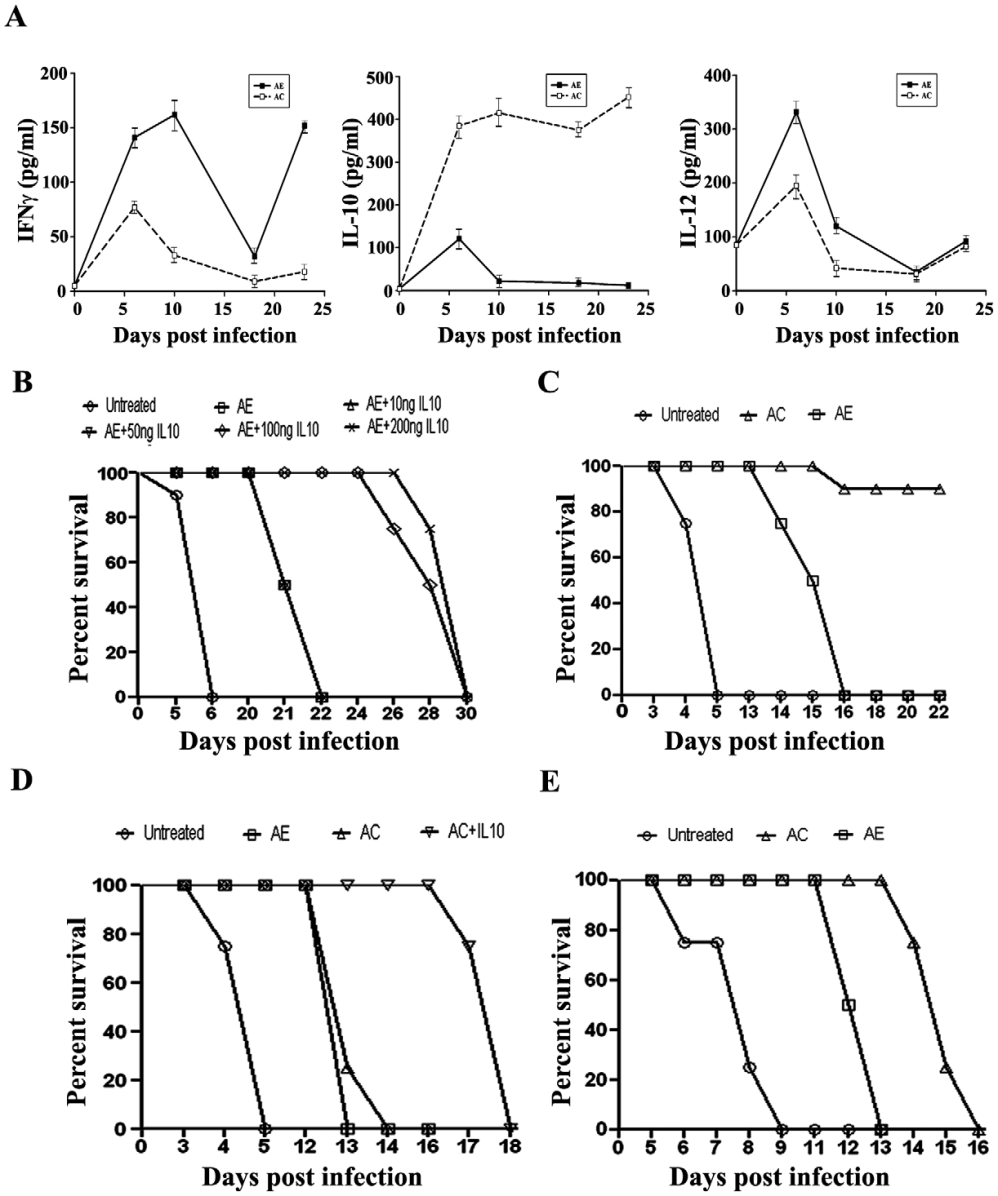
Effect of AE and AC-treatments on changes in serum cytokine levels and survival response of TLR2^−/−^ and IL-10^−/−^ mice to *P.berghei*-infection. (A) Changes in the serum levels of INFγ, IL-10 and IL-12. The data represent Mean + S.D. from three sera preparations and collected during day 6 (D6) to day 23 (D23) at intervals. The day 0 values correspond to those of uninfected animals. The values (pg/ml) corresponding to infected animals, which all died on day 6, are as follows: INFγ, 274±22; IL-10, 422±29; IL-12, 510±39. The values corresponding to infected animals treated with curcumin alone and which all died between, 7–8 days are as follows: INFγ, 243±22; IL-10, 310±33; IL-12, 377±39. (B) Effect of IL-10 injection in AE-treated animals. IL-10 was injected on alternate days from day 10 in five doses ranging from 10 ng to 200 ng. Alternate i.p. and i.v. routes were used. (C) Survival response of *P.berghei*infected C57BL/6 mice to AE and AC-treatments. The data are from two experiments with five animals in each group. (D) Survival response of TLR^−/−^ animals with and without IL-10 injection (100 ng per animal for 5 days). (E) Survival response of IL-10^−/−^ animals. Four animals were used in each group for the experiments in (B), (D) and (E). The knock-out animals were in C57BL/6 background. While, the animals were injected with around 10^4^ parasites in general, the IL 10^−/−^ animals received around 10^3^ parasites. UI, unifected; I, infected; UT, untreated; C, curcumin; AE, ART alone; AC, ART+CUR.

We next examined whether spleen mRNA levels were reflected in the serum levels of the cytokines during the course of the drug treatments. The results presented in Figure 4A indicate that there was an increase in both pro- and anti-inflammatory cytokine levels soon after infection. AC treatment suppressed the pro-inflammatory cytokine IL-12 and IFNγ levels. There was a striking increase in anti-inflammatory cytokine IL-10 levels under these conditions, correlating with protection during the recrudescence phase. IL-10 injection to AE-treated animals delayed the death of the animals (Figure 4B).

### AC Treatment Was Not Protective in *P.berghei*-Infected TLR2^−/−^ and IL-10^−/−^ Mice

It has been suggested that TLR2 agonists are specialized in inducing IL-10 expression by antigen-presenting cells (APCs). This has been shown with respect to macrophages and DCs stimulated by antigens from mycobacterium, Yersinia and pneumococcal cell wall [29]. If this were to be true in the present context, AC treatment would fail in TLR2^−/−^ animals. This study was carried out in these knock out animals (C57BL/6) with and without IL-10 injection. It needs to be pointed out that C57BL/6 animals were more sensitive to *P. berghei*-infection than Swiss mice and the recrudescence in AE treatment started earlier, although the protective effect of AC treatment was seen in this strain as well (Figure 4C). The results presented in Figure 4D indicate that AC treatment failed to protect in TLR-2^−/−^ animals and IL-10 injection was able to delay the death of the animals. These results also indicate that continuous production of IL-10 was essential for maintaining the concentration of the cytokine to offer protection. Finally, it is of interest to establish the role of IL-10 by examining the protective efficacy of AC treatment in IL-10^−/−^ animals. The results presented in Figure 4E indicate that AC treatment delayed death by a couple of days, but could not protect against recrudescence in these animals. Thus, the role of IL-10 in mediating the protective effect of curcumin against recrudescence is clearly established. A previous report indicates that maintenance of higher levels of IL-4 and IL-10 may be associated with protection of BALB/c mice from early death compared to C57BL/6 mice [26]. IL-10 has also been shown to play a crucial role in the protection of experimental cerebral malaria due to *P. berghei* ANKA strain by co-infection with non-lethal malaria parasite [30]. The recovery of 40% of recrudescing *P.berghei*-infected animals under conditions of the alkaloid manzamine A treatment has also been shown to be correlated with increased IL-10 levels in sera [31].

### AC Treatment Enhanced Anti-parasite IgG Subclass Levels in Serum

**Figure 5:**
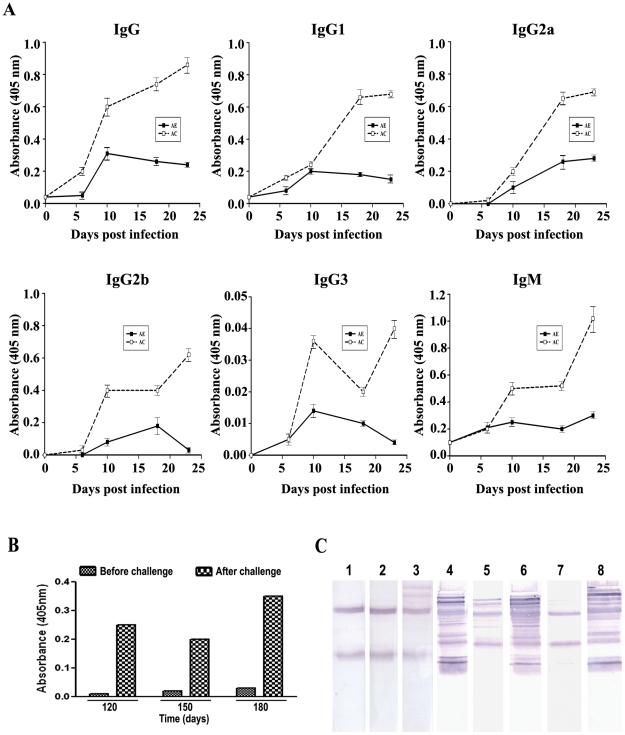
Effect of AE and AC-treatments on changes in serum total IgG and IgG-subclass antibody levels and Western analysis of parasite proteins with such sera in *P.berghei*-infected mice. Antibody levels were quantified in sera by ELISA using microtitre plates coated with parasite lysate from day 6 (D6) onwards. (A) Changes in the serum levels of total IgG and IgG-subclass antibodies against parasite lysate. The data represent Mean ± S.D. from three sera preparations (pooled from two animals each) and collected during day 6 (D6) to day 23 (D23) at intervals. The day 0 values correspond to those of uninfected animals. The values (A_405_) corresponding to infected animals, which all died on day 6, are as follows: IgG, 0.07±0.002; IgG1, 0.03±0.005; IgG2a, not detectable; IgG2b, not detectable; IgG3, not detectable; IgM, 0.22±0.007. (B) Anamnestic response of AC-treated animals. The animals were injected with fresh parasitized blood 10 days before the day mentioned in the Figure. The antibody titre (IgG) was measured before and after challenge. The data represent an average from two sera preparations. (C) Western blot analysis of parasite proteins with the different sera preparations. 1, uninfected; 2, day 5- infected; 3, AE -day 22; 4, AC day-22; 5 and 6, AC - day 75, before and after challenge; 7 and 8; AC - day 180, before and after challenge.

AC treatment resulted in a striking increase in anti-parasite antibodies encompassing all IgG subclasses, contributing to protective immunity during the recrudescence phase (Figure 5A). At later periods of time, when the antibody titre waned, anamnestic response was observed with fresh parasite injection (Figure 5B). The animals were completely protected and mice are known to be protected once cured of the malarial parasite infection [32]. These results are also corroborated by Western blot analysis, where sera from the recrudescence and later phases in AC treated animals interacted with several parasite proteins, compared to infected or AE-treated controls (Figure 5C).

Thus, quantification of TLR and cytokine mRNAs in the spleen, assessment of cytokine and IgG subclass levels in serum, response of TLR-2^−/−^ and IL-10^−/−^ animals with and without IL-10 injection to AE and AC treatments have led us to conclude that the immunomodulation brought about by AC treatment involves activation of TLR-2, followed by production of IL-10 and anti-parasite antibodies to prevent parasite recrudescence. IL-10 was initially described as a Th2 type cytokine, but subsequent studies have shown that it is expressed by many cells in the adaptive and innate immunity pathways, resulting in diverse immunological responses, including Th1 and Th2 types [28]. While, the delineated pathway is, perhaps, the central mechanism of action of curcumin in combination with ART, further studies are needed to unravel the molecular interactions, interplay between cytokines and the immune cells involved in memory response. Although, curcumin is known to have a wide variety of biochemical, pharmacological and immunological effects and is being tested as a cure against a variety of diseases ranging from Alzheimer [33], cystic fibrosis [34] to cancer in long-term studies [35], the present study uniquely positions this molecule in a 3-day therapy in combination with ART, to be tested against recrudescence and relapse in falciparum and vivax malaria. Primaquine, in a 14-day therapy, is the only anti-relapse drug available against vivax malaria and hypnozoites are considered as a hidden obstacle to malaria elimination [36]. In view of the potent anti-inflammatory effect of curcumin its potential to be tested as an adjunct therapy in cerebral malaria has also been suggested [37], [38].

## Materials and Methods

### Treatment of mice

Animal experiments were carried out as per the guidelines of the Committee for the Purpose of Control and Supervision of Experimental Animals (CPCSEA), Government of India (Registration No: 48/1999/CPCSEA) and as approved by the Institutional Animal Ethics Committee (IAEC) of the Indian Institute of Science, Bangalore (CAF/Ethics/102/2007-435 and CAF/Ethics/192/2010). The study was approved and supported by the Department of Biotechnology, New Delhi (IDs: BT/PR10541/Med/15/605/2008 and BT/01/10/MPB/DT).

All manipulations in mice were carried out under pentobarbital anesthesia and all precautions undertaken to minimize suffering. Mice (around 30 g) were injected intraperitoneally with *P. berghei* (ANKA strain) – infected mouse blood (60–65% parasitemia) on day 0 after appropriate dilution. The animals developed high parasitemia and died between 5–6 days. The artemisinin derivative α, β - arteether (ART) was injected intramuscularly on day 3 at a dose of 750 µg/mouse (single injection). This was followed by 3 oral doses of curcumin (CUR, 5 mg/mouse in DMSO) given at 24 hr intervals.

### Curcumin estimation in plasma

Extraction of curcuminoids from plasma was carried out as described by Song *et al* [13]. Briefly, the plasma samples were adjusted to pH 3.0 with 6N HCl and extracted twice with twice the volume of a mixture of ethylacetate and isopropanol (9∶1, v/v). The ethylacetate layer was concentrated in a stream of nitrogen and used for MALDITOFMS measurements as described for curcumin estimation in mouse lung cell cultures [13]. Curcumin concentration was calculated from a standard curve drawn in the range 1 to 10 ng.

### RT-PCR and Real Time PCR

Semi-quantitative RT-PCR was carried out for parasite 18S ribosomal RNA with the following primer pair to obtain a 390 bp PCR product: 5′-AGGGATGTATTCGCTTTATTTAATGC-3′ and TCTTGTCCAAACAATTCATCATATC-3′. The reaction was carried out for 30 cycles as per the following regimen: Denaturation at 95°C for 45 sec. annealing at 50°C for 40 sec. and extension at 72°C for 40 sec, with a final extension for 10 min. The RT-PCR product was analysed by electrophoresis on 1% agarose gels. Total RNA was isolated from infected red blood cells, liver and spleen by TRIZOL method (Invitrogen) followed by DNAse I treatment. 1 µg of RNA was taken for RT reaction (45°C for 60 min) using random hexamers (Pharmacia). A tenth of the product was taken for PCR reaction. Parasite load was assessed using Real Time PCR for 18S ribosomal RNA [9]. Real Time PCR (ABI Prism® 7900 HT with SDS software version 2.1.1) was carried out as per manufacturer's protocol (Finnzymes-F-410L DYNAmoTM HSSYBR Green a PCR Kit). 1.5 µg of RNA was taken for RT reaction and different concentrations of cDNA were taken for standardization. The primer pair designed to obtain a 143 bp PCR product was: 5′-GTTAAAAGAATTGACGGAAGGGCACCACCAG-3′ and 5′-TGCATCACCATCCAAGAAATCAAGAAAGAG-3′. The Ct values were calculated for the RNAs isolated from parasitized blood with different parasitemia levels (assessed by Giemsa stain and counting parasitized cells in different fields) and a correlation was established between Ct values and parasitemia (Figure S1) and the results are expressed in terms of the latter.

Semi-quantitative RT-PCR with standard primers was also carried out with RNA isolated from spleen for the TLR-, cytokine- and IgG sub-class mRNAs mentioned. A list of the primers used is given in Table S3.

### Measurement of intracellular ROS

For this purpose mice were infected with *P. berghei* for 72 hr and then received a single injection of ART and one dose of curcumin by oral feeding. Blood was collected 12 hr after drug treatment. The formation of intracellular ROS in parasitized red blood cells was measured using 2′,7′-dichlorofluorescin diacetate (DCFH-DA, Sigma-Aldrich) essentially as described by Cui *et al* [18]. Briefly, 200 µl of blood was appropriately diluted with PBS and 100 µl was incubated in presence of DCFH-DA (5 µM) for 30 min at 30°C. Excess DCFH-DA was removed by washing with PBS, suspended in 1.5 ml PBS and 50 µl analysed by FACS using caliber flow cytometer (Beckton Dickinson) equipped with an excitation laser line at 488 nm and Cell Quest software (BD). The parasitized and unparasitized erythrocytes were distinguished during the flow cytometric analysis on the basis of fluorescence intensity with the former and latter designated to different quadrants. The DCFC green fluorescence was collected in a log scale through a 530+20 band pass filter. For each analysis 10,000 events were recorded. Monoparametric histograms of the fluorescence distribution were plotted for the estimation of ROS production. The results are expressed in terms of % fluorescent cells and mean fluorescent intensity.

### Hemozoin estimation

For this purpose, mice were infected for 72 hr with *P. berghei* and then received a single injection of ART and one oral dose of curcumin. Blood was collected after 8 hr and 12 hr and parasites were isolated from red blood cells after lysis with saponin. Hemozoin content was estimated in the parasite as described by Tripathi *et al* [39], who showed that multiple washing steps are necessary to obtain hemozoin free of monomeric and bound heme. Briefly, the parasite pellet was suspended in Tris-HCl buffer containing 2.5% SDS and incubated at 37°C for 30 min and centrifuged. The pellet was suspended in alkline-bicarbonate buffer (100 mM, pH 9.2) and centrifuged. The final hemozoin pellet was hydrolysed using IN NaOH: 2.5% SDS (1∶9) and the heme released measured at 405 nm.

### Estimation of parasite-specific IgGs and cytokines in serum

Soluble parasite proteins were prepared as described by Ang *et al* [31]. The parasite pellet was suspended in an appropriate volume of PBS followed by sonication (5 min at 4°C) and centrifugation at 10,000 g for 10 min. The supernatant was stored at −80°C and used to estimate parasite – specific IgGs by ELISA. 10 µl of the supernatant was coated onto 96-well ELISA plates (4°C overnight) and reacted with 1∶10 diluted serum in a total volume of 100 µl. After carrying out the blocking and washing steps, 50 µl of secondary goat anti-mouse IgG-peroxidase conjugate (Genei TM) at 1∶3000 dilution was added per well and color developed with ABTS substrate was measured at 405 nm using an ELISA plate reader. IgG sub-class antibodies were obtained from Santa Cruz Biotechnology, Inc, USA. INF-γ, IL-10 and IL-12 were all measured in 10 µl of mouse serum using Murine ELISA Development Kits from PEPROTECH Asia (Israel) as per the manufacturer's protocol. Biotinylated Detection Antibody and Avidin-HRP conjugate were used with ABTS liquid substrate to give a colored product measured at 405 nm. The sera were also used to check for antibodies against total parasite proteins by Western blot analysis. For this purpose, the parasite pellet was suspended in SDS-buffer and 40 µg protein was analysed on SDS-PAGE (10% polyacrylamide) followed by Western analysis with different sera.

### Experiments with Knock Out animals

TLR-2^−/−^and IL-10^−/−^ mice in C57BL/6 background were the kind gifts from Prof. Ruslan Medzhitov (Yale University School of Medicine, New Haven, USA). These animals are subsequently being maintained in a specific pathogen free animal facility at International Centre for Genetic Engineering and Biotechnology, New Delhi. The protocols for infection and drug treatment were the same as for the Swiss mice described earlier, except that while around 10^4^ parasites were injected in the case of wild-type (Swiss) mice and TLR2^−/−^ (C57BL/6) animals, the IL10^−/−^ (C57BL/6) animals received around 10^3^ parasites. This resulted in a slightly longer survival period for the latter, but AC could not protect even under these conditions. In view of the paucity of IL 10^−/−^ animals, IL 10 injected group was not included. Four animals were used in each group.

### Statistics

Long-term experiments (Figures 1A, 4A and 5A) were repeated thrice and 3 animals were used per group in each experiment. The data represent Mean ± S.D. from 3 animals in one such series. The results obtained in the repetitive experiments are very similar, except that the responses varied by 1–3 days between the series. All other data are from experiments with 4 or 5 animals in each group. Sera were pooled from two mice for each measurement. Significance between control and treated group was examined using Student's *t* test and values of P<0.05 are considered as significant.

## Supporting Information

Figure S1 Correlation between Real-Time PCR of parasite 18S rRNA (Ct values) and parasitemia. RNA was isolated from the parasite and Real-Time PCR for 18S rRNA was carried out at different parasitemia values ranging from 5% to 70% as quantified using Giemsa stained blood smears.
(TIF)

Figure S2 Semi-quantitative RT-PCR analysis for parasite 18S rRNA with RNA from blood of *P. berghei*-infected mice. UT, untreated; C, curcumin; AE, ART alone; AC, ART+CUR. GAPDH -RNA was used as loading control.
(TIF)

Table S1 Comparative effects of AE and AC Treatments on the survival of *P. berghei*-infected mice. The data provided are from 20 experiments carried over a period of 4 years. The animals were infected with *P. berghei* for 72 hr and then given a single injection of ART (AE), followed by three oral doses of curcumin (AC) at 24 hr intervals. The experimental details are given in the main paper.
(DOC)

Table S2 Curcumin (C) and Demethoxy Curcumin (DC) concentrations in mice tissues. Data represent Mean ± SD from three preparations. ^*^µg/ml; ^#^µg/g; ND: not detectable.
(DOC)

Table S3 Primers used for semi-quantitative RT-PCR analysis of RNA from spleen. Primers were designed for the IgG sub-class antibodies and unique forward primers and a common reverse primer were used. The products obtained were verified and characterized based on unique restriction sites. Standard primers were used in other cases.
(DOC)
