# Supplementary figures and images for: Curcumin-Arteether Combination Therapy of Plasmodium berghei-Infected Mice Prevents Recrudescence Through Immunomodulation
Source: PLoS One. 2012 Jan 20;7(1):e29442. doi: 10.1371/journal.pone.0029442 (PMC3262785; doi:10.1371/journal.pone.0029442)

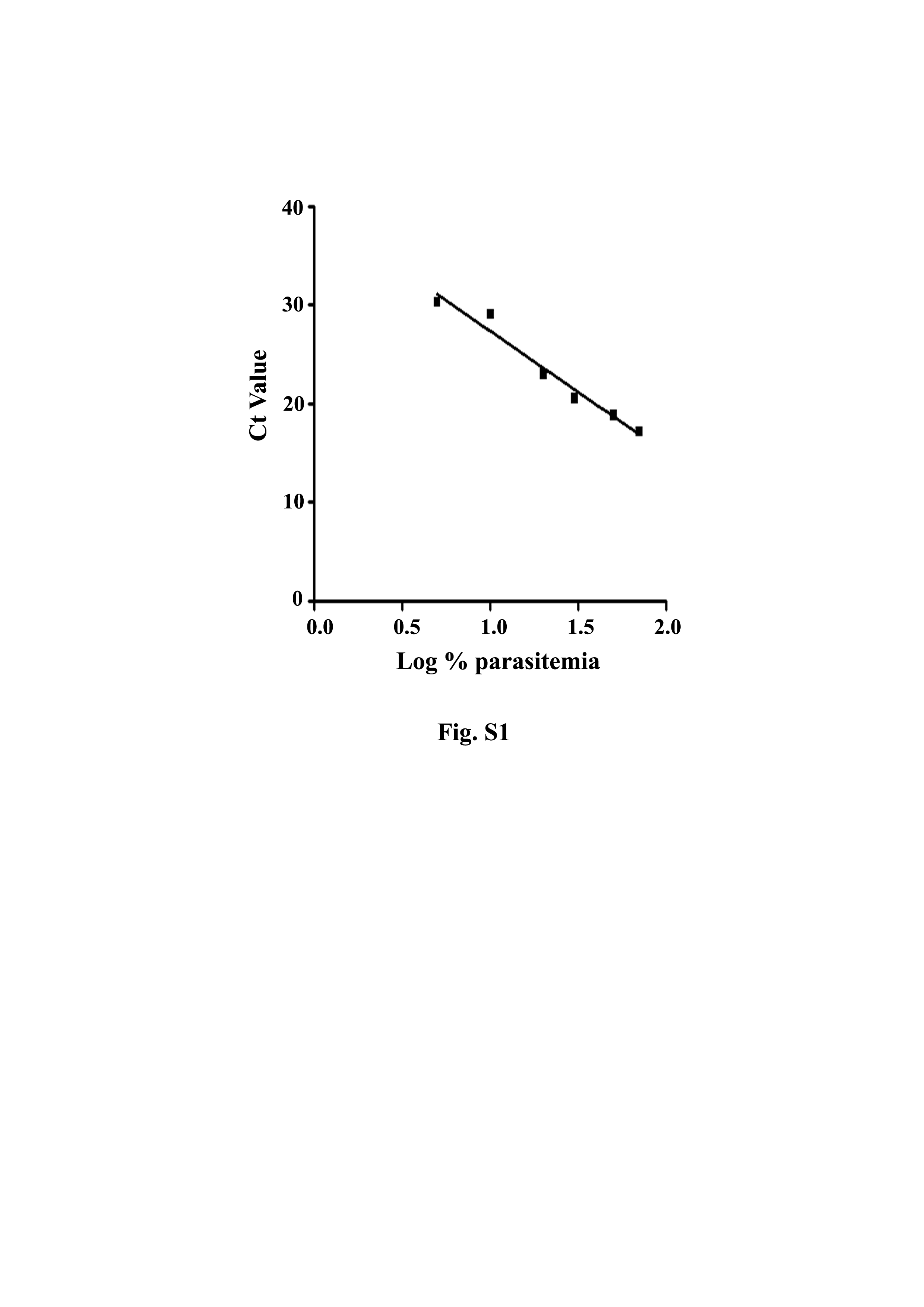

Supplement: Figure S1 — Correlation between Real-Time PCR of parasite 18S rRNA (Ct values) and parasitemia. RNA was isolated from the parasite and Real-Time PCR for 18S rRNA was carried out at different parasitemia values ranging from 5% to 70% as quantified using Giemsa stained blood smears. (TIF) [file pone.0029442.s001.tif]

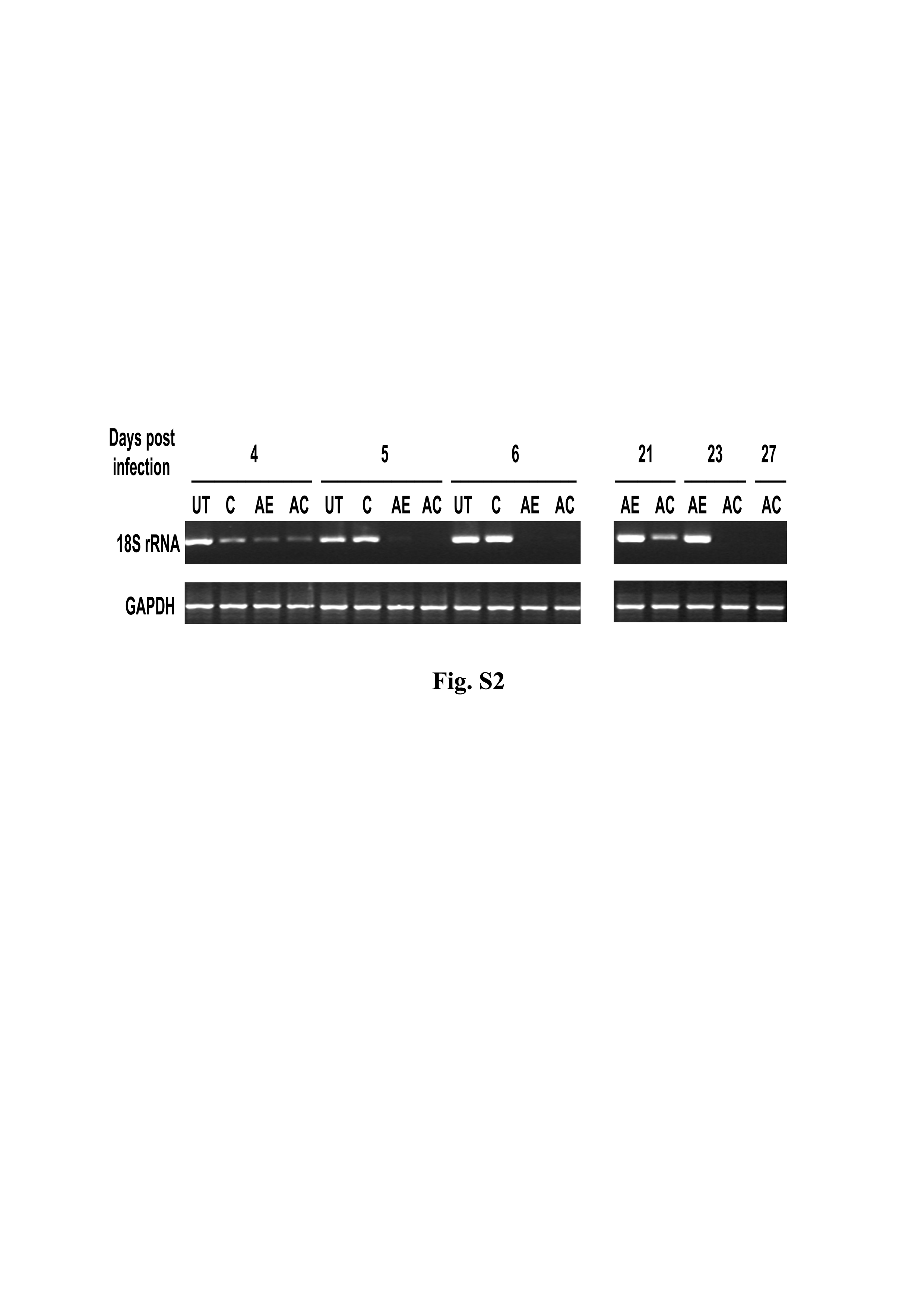

Supplement: Figure S2 — Semi-quantitative RT-PCR analysis for parasite 18S rRNA with RNA from blood of P. berghei-infected mice. UT, untreated; C, curcumin; AE, ART alone; AC, ART+CUR. GAPDH -RNA was used as loading control. (TIF) [file pone.0029442.s002.tif]
