# Supplementary material for: Curcumin-Arteether Combination Therapy of Plasmodium berghei-Infected Mice Prevents Recrudescence Through Immunomodulation
Source: PLoS One. 2012 Jan 20;7(1):e29442. doi: 10.1371/journal.pone.0029442 (PMC3262785; doi:10.1371/journal.pone.0029442)
Supplement: Table S1 — Comparative effects of AE and AC Treatments on the survival of P. berghei-infected mice. The data provided are from 20 experiments carried over a period of 4 years. The animals were infected with P. berghei for 72 hr and then given a single injection of ART (AE), followed by three oral doses of curcumin (AC) at 24 hr intervals. The experimental details are given in the main paper. (DOC) [file pone.0029442.s003.doc]

Table S1. Comparative effects of AE and AC Treatments on the survival of *P. berghei*-infected mice.

| Period | Treatment | No. of animals used | No. of animals survived | | | |
| --- | --- | --- | --- | --- | --- | --- |
|  |  |  | Days 5-8 | Days 22-25 | Day 180 | % Survival |
| 2006 -2010 | Control (Infected) | 94 | 5 | 3 | 3 | 3.0 |
|  | AE-treated | 192 | 189 | 9 | 7 | 3.6 |
|  | AC-treated | 210 | 204 | 200 | 199 | 94.7 |
