# Supplementary material for: Curcumin-Arteether Combination Therapy of Plasmodium berghei-Infected Mice Prevents Recrudescence Through Immunomodulation
Source: PLoS One. 2012 Jan 20;7(1):e29442. doi: 10.1371/journal.pone.0029442 (PMC3262785; doi:10.1371/journal.pone.0029442)
Supplement: Table S2 — Curcumin (C) and Demethoxy Curcumin (DC) concentrations in mice tissues. Data represent Mean ± SD from three preparations. *µg/ml; #µg/g; ND: not detectable. (DOC) [file pone.0029442.s004.doc]

Table S2. Curcumin (C) and Demethoxy Curcumin (DC) concentrations in mice tissues.

_____________________________________________________________________________________________

Uninfected Infected

Treatment _____________________________________ ____________________________________

Plasma^*^ RBC^*^ Liver ^#^  Spleen^#^ Plasma^*^ RBC^*^ Liver ^#^  Spleen^#^

_____________________________________________________________________________________________

A. Curcumin

C: 2.10±1.21 1.20±0.92 0.78±0.42 2.40±1.21 1.56±0.76 0.62±0.32 2.05±0.82 1.62±0.45 DC: ND 0.74±0.21 1.10±0.82 2.50±1.21 ND 0.29±0.12 0.89±0.42 1.56±0.82

B. AE + Curcumin

C: 1.47±0.62 0.75±0.35 2.30±0.91 2.91±1.46 2.36±1.22 0.66±0.45 3.20±1.52 2.96±1.26

DC: ND 0.61±0.42 1.26±0.53 1.31±0.82 ND 0.40±0.25 1.59±0.72 1.31±0.55

_____________________________________________________________________________________________
