# Supplementary material for: Curcumin-Arteether Combination Therapy of Plasmodium berghei-Infected Mice Prevents Recrudescence Through Immunomodulation
Source: PLoS One. 2012 Jan 20;7(1):e29442. doi: 10.1371/journal.pone.0029442 (PMC3262785; doi:10.1371/journal.pone.0029442)
Supplement: Table S3 — Primers used for semi-quantitative RT-PCR analysis of RNA from spleen. Primers were designed for the IgG sub-class antibodies and unique forward primers and a common reverse primer were used. The products obtained were verified and characterized based on unique restriction sites. Standard primers were used in other cases. (DOC) [file pone.0029442.s005.doc]

Table S3. Primers used for semi-quantitative RT-PCR analysis of RNA from spleen.

| IL-10 | 5'-GTGAAGACTTTCTTTCAAACAAAG-3'  5'-CTGCTCCACTGCCTTGCTCTTATT-3' |
| --- | --- |
| TGF–β | 5'-CTGGGACCCTGCCCCTATAT-3'  5'-GGGCAAGGACCTTGCTGTAC-3' |
| IL-12 | 5'-ACGCAGCACTTCAGAATCACA-3'  5'-CACCAGCATGCCCTTGTCTA-3' |
| IFNγ | 5'-ACGGCACAGTCATTGAAAGCCTA-3’  5'- GTCACCATCCTTTTGCCAGTTCC-3' |
| IgG1 | 5'-GGGATTGTGGTTGTAAGCCTTGCATATGTACAG-3' |
| IgG2A | 5'-CACAATCAAGCCCTGTCCTCCATGCAAATG-3' |
| IgG2B | 5'-ATTTCAACAATCAACCCCTGTCCTCCATGCAAG-3' |
| IgG3 | 5'-CAAGCCCAGTACCCCCCCAGGTTCTTC-3' |
| Common Reverse Primer for IgGs | 5'-GTTGACCTTGCATTTGAACTCCTTGCC-3' |
| GAPDH | 5'- ATGGTGAAGGTCGGTGTGAACGGA-3'  5'- TTACTCCTTGGAGGCCATGTAGG-3' |
| TLR2 | 5'-CTCCTGAAGCTGTTGCGTTAC-3'  5'-CTACTGTGATTCGCTTCACCTTC-3' |
| TLR4 | 5'-CAAGGGGTTGAAGCTCAGAT-3'  5'-TTCTTCTCCTGCCTGACACC-3' |
| TLR9 | 5'-GGCTTCAGCTCACAGGGTAG-3'  5'-GAATCCTCCATCTCCCAACA-3' |
